# The effect of serving as a danwei leader before retirement on self-rated post-retirement health: empirical evidence from China

**DOI:** 10.1186/s12889-022-12937-z

**Published:** 2022-03-23

**Authors:** Li He, Kun Wang, Jiangyin Wang, Zixian Zhang, Yuting Wang, Tianyang Li, Yuanyang Wu, Shuo Zhang, Siqing Zhang, Hualei Yang

**Affiliations:** 1 School of Philosophy, Zhongnan University of Economics and Law, Wuhan, Hubei China; 2 School of Public Administration, Zhongnan University of Economics and Law, No. 182, Nanhu Avenue, Donghu New Technology Development Zone, Wuhan, Hubei China

**Keywords:** Chinese danwei leaders, Retirement, Occupation, Self-rated health, China longitudinal aging social survey

## Abstract

**Background:**

Worker health is often influenced by their occupation type, and the accumulative effect of occupation has a significant impact on their health after retirement. Studies show that the type and level of occupation before retirement directly impact workers’ self-rated post-retirement health. However, there is little research on the self-rated post-retirement health of danwei leaders in China. This study seeks to examine the self-rated health level of Chinese danwei leaders after retirement. Furthermore, the differences between their self-rated health level and that of retirees from other occupations are explored by examining the accumulative effect of occupation and the mechanism underlying these differences.

**Methods:**

Based on 5,910 samples’ data from the 2018 China Longitudinal Aging Social Survey, ordinary least squares, logit, and propensity score matching models are used to investigate the self-rated health level of Chinese danwei leaders after retirement, their differences with other occupations, and the corresponding mechanism.

**Results:**

The results show that retired danwei leaders have higher self-rated health levels than retirees in other occupations. This was mainly found among female, non-eastern, married, not living with children, and highly educated respondents. The difference in self-rated health between retired danwei leaders and other retired groups was influenced by their post-retirement income and social status.

**Conclusions:**

In China, retired danwei leaders rated their health higher than retirees from other occupations. Danwei leaders have high socioeconomic status due to their occupation. Compared with other groups, their advantages are significant and enjoyed for a long time. Additionally, most danwei leaders have high social influence even after retirement and their higher income and social status have a positive impact on their self-rated health compared with other employees. This study provides empirical evidence from China and extends current literature on the effects of occupational type and level on self-rated health after retirement.

## Introduction

Research on workers’ health needs to consider the role of occupational type. Since the impact of occupation on health is accumulated throughout life [1], this accumulative effect of the impact of occupation on the health of older adults is particularly important [2]. Therefore, this study focuses on the self-rated health of retired Chinese *danwei* leaders by examining the accumulative effect of occupation.

Danwei is a form of social organization in Chinese culture that oversees many public affairs such as housing, medical care, pension, and education, among others. It originally referred to the working units of government agencies or official organizations and later expanded to all types of public sectors, including party and government agencies, enterprises, education and research institutions, and government-funded social organizations, etc. [3]. The leader of a danwei in China refers to the person in charge or senior management of the above public sectors. This means that they have an advantage over ordinary employees in other occupations and lower levels. Chinese danwei leaders often have great authority and social influence; they also enjoy higher incomes and social status. This advantage is long term and can continue to accumulate, which will have an impact on their health after retirement. This raises the question, “Compared with retirees in other occupations, will the health status of the danwei leaders after retirement be better due to the advantages of their occupation and the accumulation of these advantages?”

As self-rated health is considered a global measure of health status [4], this study attempts to examine whether retired danwei leaders have a higher self-rated health level than other occupational retirees. There are at least two contributions of research on this issue. From a theoretical perspective, research on the impact of the pre-retirement occupational level and status on post-retirement health in China will enrich international theoretical and empirical research on retirement and health as well as occupational level and health. From a practical perspective, it can provide evidence for China to formulate differentiated pension policies for diverse occupational retirement groups; further, it can act as a reference for people making career choices. Additionally, this study is of great significance to the all-round development of society.

## Literature review

In recent years, research on retirement and health issues in China has gradually unfolded. Chinese danwei leaders have attracted the attention of an increasing number of Chinese scholars because of their professional nature and other particularities; however, most of their research focuses on specific professions such as senior managers in state-owned enterprises [5, 6] and senior government officials [7]. Moreover, the literature on the retirement and health of danwei leaders is limited. As far as we know, only one study has focused on danwei leaders’ life satisfaction after retirement. Based on CLDS data, He et al. found that danwei leaders have higher life satisfaction after retirement compared to other occupational groups [8]. However, the authors did not mention the health status of danwei leaders after retirement. Compared with China, international research on retirement and health has formed a relatively complete and independent system. Studies on the relationship between retirement and physical, psychological, and self-rated health have been sufficient; further, studies on factors affecting post-retirement health (e.g., sex, education level, family status, occupational type, and income) have also been very comprehensive [9–11]. Many studies have been conducted on the interaction between retirement and health.

Self-rated health is a valid indicator of physical and mental health [12]. In terms of the relationship between retirement and self-rated health, some existing research conclusions are controversial. Retirement is considered a major life change, which often brings pressure [13]. In particular, using panel data from 11 European countries and two distinct identification strategies, Hellersahlgren [14] found that long retirements resulted in lack of work, which had a robust negative effect on self-rated health. Retirement changes socioeconomic status and lifestyles, resulting in lower socioeconomic status, less physical activity, and less social interaction. This leads to a 6–9% decrease in mental health and a 5–6% increase in the risk of disease [15], especially a significant increase in the risk of chronic diseases such as serious cardiovascular disease and cancer. These factors all worsen self-rated health [16]. While some scholars have found that retirement has no significant effect on self-rated health [17], many others have shown that retirement has a positive effect. On the one hand, by analyzing the self-rated health of French GAZEL employees before and after retirement, Westerlund et al. [18] found that retirement was related to a significant reduction in mental and physical fatigue and depressive symptoms. This is not only because retirement can keep people away from mentally or physically stressful work environments [19], but also because the time reallocated due to retirement leads to increased sleep [20]. On the other hand, retirement provides more opportunities to be physically active [21], especially moderate-intensity physical activities [22]. Under the influence of these factors, most retirees would have a better self-perception of health, that is, a higher subjective health assessment [23]. Thus, retirement can greatly improve an individual’s self-rated health [24]. However, the conclusion that retirees are more physically active and pursue healthier lifestyles to achieve better self-rated health has also been questioned. Johnston and Lee [25] found that this is only an assumption and not necessarily true. They showed that the difference in individuals’ assessment of their own health status after retirement may be due to the change in their reference group.

The influencing factors of the relationship between retirement and self-rated health have also been discussed. Sex, family status, education level, and occupational type have different effects on the self-rated health level of retirees [26–28], with occupational factors having a particularly significant impact [2]. Existing studies have also focused on the effect of pre-retirement occupation type on workers’ post-retirement self-rated health. Different occupation types generally have varied living standards and health statuses, making their self-rated health different across types. Studies have shown an association between low income and higher rates of ill-health [29–33] because manual workers with lower incomes and occupational levels have suffered serious negative occupational effects due to poor working conditions [34] and greater physical harm from work [35]. This negative effect is long-lasting, continuous, and gradually accumulated, which lowers their self-rated health level after retirement. However, although blue-collar workers have a higher incidence of disease after retirement [36], Tuomi et al. [37] found no clear evidence that employees with manual jobs are more adversely affected by retirement than those with mentally challenging or mixed jobs. Meanwhile, Rose [24] proposed the opposite view: For people in low-end jobs, especially manual workers, the positive impact of retirement is very significant; being away from work is conducive to the recovery of their physical health and will improve the self-rated health level among this group.

There are different views on the self-rated health of people with lower occupational status after retirement, while studies on people with higher occupational status are generally more consistent. In general, money affects health [38]. Older adults of lower socioeconomic status have a higher risk of poor health than those of higher socioeconomic status [39–44]. This is because those with higher socioeconomic status are free to decide when to leave the labor market; retirement is a conscious positive choice and a healthy investment. Since their self-management skills and abilities are higher and they are more likely to engage in healthy activities after retirement, retirement positively impacts their self-rated health [45]. For example, white-collar workers have a certain degree of security in their retirement life. Their work requires high intelligence, low physical constraints, and a high opportunity cost of exercise. However, after retirement, the mental demands of work and the opportunity cost of exercise disappear, their stress and burdens are reduced, and the motivation to exercise increases, which helps them better able to self-manage their bodies. Thus, retirement has a protective effect on health [46], and self-rated health is naturally higher. Furthermore, owing to their relatively high pre-retirement income, industrial and service workers effectively adapt to retirement and have a high self-rated health level [47].

As a group with higher socioeconomic status, Chinese danwei leaders enjoy better living conditions and medical standards, before retirement than people in most other occupations. However, it is yet to be examined whether the long-term accumulation of this advantage will have a positive impact on their life after retirement. Further, the mechanism underlying their higher self-rated health level is yet to be investigated. From the aforementioned literature review, we can see that the studies in this area are insufficient. In particular, there is a lack of research on the impact of occupational types and levels on health after retirement. Hence, this study attempts to explore the differences in self-rated health levels between retired danwei leaders and retirees in other occupations as well as the mechanism behind these differences.

Compared with previous studies, this study adopts Chinese samples to verify existing research. It adapts the sample to China by using the ordinary least squares (OLS) model to investigate the impact of danwei leader status before retirement on self-rated post-retirement health and analyze the influencing mechanism. The specific variables assessed were self-rated post-retirement health and pre-retirement leadership status.

## Method

### Data

The data used in this study were obtained from the 2018 China Longitudinal Aging Social Survey (CLASS), which was organized and implemented by Renmin University of China. The survey targets Chinese citizens who are over 60 years old and uses a stratified multistage probability sampling method to systematically collect the socioeconomic background data of Chinese older adults, providing an important factual basis for evaluating the actual effects of various policies and measures on the quality of life of these older adults. According to the research questions and research needs, this study uses older adults who are no longer engaged in income-producing jobs (i.e., retired older adults) as the research participants. After excluding samples with missing data, refusal to answer, and obvious errors in key variables, the effective analysis sample in this study was 5,910. Table 1 shows the means of the main variables before and after data exclusion. It can be found that the screened samples are very close to the overall sample in terms of the means and standard deviations (SDs) of the main variables, indicating that the data screening process does not have much impact on the final estimation results.

**Table 1:** Comparison of different samples

Sample	Stat.	Health	Occupation	Sex	Age	Education
Total	Mean	3.282	0.052	0.481	72.220	2.924
	SD	0.902	0.223	0.500	7.539	1.309
	Min	1	0	0	52	1
	Max	5	1	1	108	5
Included	Mean	3.317	0.061	0.504	71.708	3.085
	SD	0.864	0.240	0.500	7.379	1.295
	Min	1	0	0	60	1
	Max	5	1	1	101	5
Excluded	Mean	3.204	0.033	0.430	73.354	2.570
	SD	0.977	0.178	0.495	7.765	1.268
	Min	1	0	0	52	1
	Max	5	1	1	108	5

*SD* Standard Deviation

### Measures

#### Dependent variable

The dependent variable in this study is self-rated health, which is obtained from the question, “How do you feel about your current physical health?” in the CLASS. The answers to this question are divided into five categories: “very healthy,” “relatively healthy,” “average,” “relatively unhealthy,” and “very unhealthy.” They were assigned 5, 4, 3, 2, and 1 points, respectively. Higher scores indicate better self-rated health.

#### Independent variable

The CLASS data provide detailed information about the respondents’ occupations before retirement. The independent variable used in this study was whether or not one is a danwei leader, based on the answer to the following CLASS question: “What did you do before you stopped the income-generating work/activity?” In terms of variable assignment, individuals who answered “state, enterprise, and public danwei leaders” were assigned a value of 1, whereas those who did not were assigned a value of 0.

#### Control variables

Based on the research question and referring to the research of Marchand [48], this study selected sex, age, education, ethnic, religious belief, marital status, offspring, living situation, social support, basic activity ability, daily activity ability, hospitalization, and region as control variables.

#### Mediating variable

According to Janzen et al. [49] and Osborn et al. [50], whether or not one serves as a danwei leader before retirement impacts an individual’s self-rated post-retirement health, mainly through two channels: post-retirement income and social status. Therefore, in this study, the mediating variables of post-retirement income and social status are used to analyze the influencing mechanism of individuals’ pre-retirement leadership on post-retirement self-reported health. We speculated possible explanations for this. First, Chinese danwei leaders can enjoy good welfare after retirement and relatively good living conditions, as well as receive a large monthly pension, thus enabling them to maintain relatively high self-rated health levels. Second, compared with other retirement groups, retired danwei leaders have higher social status; not only are their life control and social participation stronger, but they also have greater social influence. Therefore, they are respected and valued, and their self-rated health is better.

Post-retirement income was obtained from the response to the question, “What is your total personal income in the past 12 months?” and is log transformed. Social status was obtained using the answer to the question, “Did you participate in dispute mediation in the past year?” In Chinese culture, people who can mediate disputes usually have good social reputation and are well respected; in other words, they are people with high social status. Therefore, to measure the social status of the retired group, this study examined whether the respondent mediated disputes in the past year and assigns a value of 1 to individuals who mediated disputes and a value of 0 to those who did not.

### Model selection

#### Ordinary least squares model

To explore whether the self-rated health of retired danwei leaders was higher than that of other occupational retirement groups, we first used the OLS model to perform a preliminary regression. The model was set as follows:

1 $$healt{h}_i={\alpha}_0+{\alpha}_1 occupatio{n}_i+{\alpha}_2{X}_i+{\varepsilon}_i$$

where *health*_*i*_ represents the self-rated health of the i-th respondent;*occupation*_*i*_ represents whether the i-th respondent is a danwei leader; *X*_*i*_ represents the other control variables;*ε*_*i*_ is the random error term; and *α*_1_ is the coefficient to be estimated in this study, which reflects the direction and magnitude of the influence of pre-retirement danwei leader status on self-rated post-retirement health.

#### Ordinal logit models

Considering that self-rated health is a typical ordinal variable, this study also used an ordinal logit model for the regression. The model was set as follows:

2 $$healt{h}_i^{\ast }=\beta occupatio{n}_i+{\delta X}_i+{\varepsilon}_i$$

where $healt{h}_i^{\ast }$ denotes an individual’s self-rated health; *occupation*_*i*_ denotes whether the individual was a danwei leader before retirement;*X*_*i*_ denotes the other control variables; and *ε*_*i*_ is the random error term. When $healt{h}_i^{\ast }$ is less than *C*_1_, the older adults perceive themselves as very unhealthy (*health*_*i*_=1). When $healt{h}_i^{\ast }$ is greater than *C*_1_ but less than *C*_2_, the older adults perceive themselves as relatively unhealthy (*health*_*i*_=2). When $healt{h}_i^{\ast }$ is greater than *C*_2_ but less than *C*_3_, the older adults perceive their health status as average (*health*_*i*_=3). When $healt{h}_i^{\ast }$ is greater than *C*_3_ but less than *C*_4_, the older adults consider themselves to be relatively healthy (*health*_*i*_=4). When $healt{h}_i^{\ast }$ is greater than *C*_4_, the older adults consider themselves as very healthy (*health*_*i*_=5). The specific settings are as follows:

3 $${health}_i=\left\{\begin{array}{c}1, healt{h}_i^{\ast}\le {C}_1\\ {}2,{C}_1< healt{h}_i^{\ast}\le {C}_2\\ {}3,{C}_2< healt{h}_i^{\ast}\le {C}_3\\ {}4,{C}_3< healt{h}_i^{\ast}\le {C}_4\\ {}5, healt{h}_i^{\ast }>{C}_4\end{array}\right.$$

#### Propensity score matching model

To solve the endogeneity problem caused by sample selection bias and the omission of explanatory variables, this study adopted the propensity score matching (PSM) method. The PSM method was based on the “counterfactual inference model” [51], which can effectively solve the problem of endogeneity. The specific research steps are as follows.

The first step is to use the logit regression model to calculate the propensity score, that is, the PS value:

4 $$\mathrm{PS}\left(\mathrm{X}\right)=\Pr \left\{\mathrm{D}=1\ |\ \mathrm{X}\right\}=\mathrm{E}\left\{\mathrm{D}|\ \mathrm{X}\right\}$$

where D is the dummy variable for whether the individual was a danwei leader before retirement. If the individual was a danwei leader before retirement, then D=1; otherwise, D=0. X represents the covariates that affect whether an individual is a danwei leader before retirement.

The second step is to match the treatment group with the control group based on propensity scores. The matching methods selected in this study were nearest neighbor matching, kernel matching, and radius matching.

The third step is to calculate the average treatment effect on the treatment group (ATT):

5 $$ATT=E\left( healt{h}_1|D=1\right)-E\left( healt{h}_0|D=0\right)$$

where *health*_1_ represents the self-rated health score of the retired danwei leaders; *health*_0_ represents the self-rated health score of individuals who were not danwei leaders before retirement; and ATT is the difference between the self-rated health score of the retired danwei leaders and the self-rated health score of other retired individuals.

#### Mediating effect model

To investigate whether the impact of serving as a danwei leader before retirement on the self-rated post-retirement health of individuals was reflected in post-retirement income and social status, according to the mediating effect test procedure proposed by Wen et al. [52], the mediating effect model of this study was built in three steps. First, we regressed the dependent variable on the core independent variable, as shown in formula (6), where *health*_*i*_ is the self-rated health level of the older adults and *occupation*_*i*_ is whether the individual was a danwei leader prior to retirement. Second, we regressed the mediating variables on the core independent variables, as shown in formula (7), where M represents the mediating variables. Third, we regressed the dependent variables on the core independent variable and mediating variables at the same time, as shown in formula (8). The mediating effect model in this study is composed of the following equations:

6 $$healt{h}_i={\alpha}_1+{\beta}_1 occupatio{n}_i+{\delta}_1{X}_i+{\varepsilon}_i$$

7 $$M={\alpha}_2+{\beta}_2 occupatio{n}_i+{\delta}_2{X}_i+{\varepsilon}_i$$

8 $$healt{h}_i={\alpha}_3+{\beta}_3 occupatio{n}_i+\gamma M+{\delta}_3{X}_i+{\varepsilon}_i$$

## Results

### Descriptive statistics

Table 2 presents the descriptive statistics of the sample data. The overall health level of the sample was relatively high, with an average self-rated health score of 3.317. The proportion of retired danwei leaders was not high, at only 6.1%. In terms of other characteristics, the average age of the sample was 71.7 years old, of which 50.4% were men. Among the retired individuals, most belonged to the Han, 76.5% had a spouse, and 36.8% lived with their children. Based on this, we compared danwei leaders with other retirement groups on a number of key variables. Table 3 shows that danwei leaders have higher mean self-rated health scores, are more likely to be men, have more years of education on average, a higher mean income, and a higher likelihood of mediating disputes than the members of other retirement groups.

**Table 2:** Variable definitions and descriptive statistics

Variable	Variable definition	Obs.	Mean	SD	Min	Max
Self-rated health	Very healthy=5, relatively healthy=4, average=3, relatively unhealthy=2, very unhealthy=1	5910	3.317	0.864	1	5
Occupation	Retired danwei leader=1, other retired individuals=0	5910	0.061	0.240	0	1
Sex	Male=1, female=0	5910	0.504	0.500	0	1
Age	The actual age of the respondent	5910	71.708	7.379	60	101
Education	Illiterate=1, old-style private school=2, elementary school=3, junior high school=4, high school and above=5	5910	3.085	1.295	1	5
Ethnic	Han ethnicity=1, minority ethnicity=0	5910	0.955	0.207	0	1
Religious belief	Has religious beliefs=1, has no religious beliefs=0	5910	0.065	0.247	0	1
Marital status	Has spouse=1, has no spouse=0	5910	0.765	0.424	0	1
Offspring	Number of surviving children	5910	2.463	1.334	1	10
Living situation	Living with children=1, not living with children=0	5910	0.368	0.482	0	1
Social support	The social support scale contains six questions, including “At least how many family members/relatives can you meet or contact in a month?" Each question is answered on a five-point scale. The higher the score, the stronger the social support.	5910	13.7618	5.239	0	30
Basic activity ability	Basic activity ability, has ability barrier=1, has no ability barrier=0	5910	0.289	0.453	0	1
Daily activity ability	Daily activity ability, has ability barrier=1, has no ability barrier=0	5910	0.198	0.399	0	1
Hospitalization	"Have you been hospitalized in the past two years?" yes=1, no =0	5910	0.290	0.454	0	1
Region	Eastern province=1, other province=0	5910	0.443	0.497	0	1
lncome	Logarithm of income in the past year	5910	8.508	1.365	4.248	12.899
Mediation of disputes	Yes=1,no=0	5910	0.213	0.409	0	1
Study	Completely non-conforming=1, comparatively non-conforming=2, general=3, comparatively conforming=4, completely conforming=5	5587	2.821	1.108	1	5

*SD* Standard Deviation

**Table 3:** Comparison of danwei leaders with other retirees

Occupation	Stat.	Health	Sex	Education	lnincome	Mediation of disputes
Other retirees	Mean	3.306	0.495	3.031	8.439	0.209
	SD	0.860	0.500	1.292	1.349	0.406
	Min	1	0	1	4.248	0
	Max	5	1	5	12.899	1
Danwei leaders	Mean	3.485	0.639	3.912	9.562	0.281
	SD	0.920	0.481	1.037	1.163	0.450
	Min	1	0	1	5.991	0
	Max	5	1	5	12.388	1

*SD* Standard Deviation

### Benchmark regression

Table 4 lists the benchmark regression results. Model 1 is an OLS regression and Model 2 is an ordinal logit regression. From Model 1, as shown in Table 4, it can be found that compared with other retirement groups, the self-rated health of retired danwei leaders is 0.117 points higher, and this result is significant at the 5% level. The result of Model 2 was consistent with that of Model 1. Considering that both models showed that higher scores indicate better the self-rated health, serving as a danwei leader had a significant effect on an individual’s self-rated health after retirement.

**Table 4:** Benchmark regression results

Variable	Model 1	Model 2
	OLS	Ologit
Occupation	0.117^a^	0.334^b^
	(0.048)	(0.119)
Sex	0.017	0.041
	(0.021)	(0.051)
Age	-0.002	-0.005
	(0.002)	(0.004)
Education	0.020 ^a^	0.063 ^b^
	(0.009)	(0.022)
Ethnic	-0.287^c^	-0.747 ^c^
	(0.047)	(0.114)
Religious belief	0.089	0.251 ^a^
	(0.046)	(0.110)
Marital status	0.073 ^a^	0.180 ^b^
	(0.029)	(0.068)
Offspring	-0.021 ^a^	-0.046 ^a^
	(0.010)	(0.023)
Living situation	-0.012	-0.032
	(0.024)	(0.058)
Social support	0.002	0.008
	(0.002)	(0.005)
Basic activity ability	-0.305 ^c^	-0.721 ^c^
	(0.028)	(0.065)
Daily activity ability	-0.368 ^c^	-0.853 ^c^
	(0.033)	(0.075)
Hospitalization	-0.279 ^c^	-0.673 ^c^
	(0.025)	(0.060)
Region	0.145 ^c^	0.317 ^c^
	(0.022)	(0.053)
_cons	3.836 ^c^	
	(0.143)	
cut1		-5.208 ^c^
		(0.351)
cut2		-3.029 ^c^
		(0.343)
cut3		-0.869 ^b^
		(0.342)
cut4		1.933 ^c^
		(0.344)
N	5910	5910
r2/pr2	0.165	0.070

Robust standard errors are in parentheses; ^a^, ^b^, and ^c^ indicate significance at the 5%, 1%, and 0.1% levels, respectively

Regarding other control variables, higher level of education indicated better self-rated health of retired older adults. Compared with other ethnic groups, the self-rated health of retired Han people was significantly lower. The self-rated health of retired older adults with spouses was significantly higher, whereas that of retired older adults with basic mobility impairment, daily mobility impairment, or hospitalization in the past two years was significantly lower. Compared with retired older adults in non-eastern provinces, retired older adults in eastern provinces had significantly better self-rated health.

### Robustness test

To control for the selection bias of the retired older adult sample, this study selected the PSM method to estimate the net effect of the self-rated health impact of being a danwei leader before retirement on an individual after retirement. It used three methods: nearest neighbor matching, kernel matching, and radius matching. Table 5 presents the results of the balance tests. It can be seen that the SDs of most variables after matching was less than 5% and that the difference between the treatment and control groups was not significant. This shows that using PSM largely eliminated the sample selection bias.

**Table 5:** Balance test results

Variable	Unmatched	Mean		Bias%	Reduced	*T*-value	*P*-value
	Matched	Treated	Control		Bias%		
Sex	U	0.639	0.495	29.4		5.33	0.000
	M	0.639	0.620	3.9	86.6	0.54	0.591
Age	U	73.32	71.602	22.7		4.30	0.000
	M	73.32	73.631	-4.1	81.9	-0.53	0.594
Education	U	3.912	3.031	75.2		12.73	0.000
	M	3.912	3.890	1.9	97.5	0.29	0.773
Ethnic	U	0.959	0.955	1.9		0.35	0.727
	M	0.959	0.959	0.0	100.0	-0.00	1.000
Marital status	U	0.807	0.762	10.9		1.95	0.051
	M	0.807	0.796	2.7	75.4	0.37	0.710
Religious belief	U	0.050	0.066	-7.1		-1.24	0.215
	M	0.050	0.058	-3.5	50.1	-0.49	0.622
Offspring	U	2.309	2.473	-12.5		-2.28	0.023
	M	2.309	2.388	-6.1	51.5	-0.85	0.397
Social support	U	14.457	13.716	14.8		2.61	0.009
	M	14.457	14.813	-7.1	52.0	-0.94	0.348
Hospitalization	U	0.331	0.287	9.4		1.77	0.076
	M	0.331	0.306	5.4	43.1	0.72	0.474
Living situation	U	0.355	0.368	-2.7		-0.50	0.621
	M	0.355	0.366	-2.3	14.8	-0.31	0.758
Region	U	0.399	0.445	-9.3		-1.70	0.089
	M	0.399	0.372	5.6	39.9	0.76	0.446

Meanwhile, Table 6 reports the average treatment effect of the self-rated health of retired danwei leaders. The nearest neighbor matching results showed that compared with the older adults who were not danwei leaders before retirement, the self-rated health score of retired danwei leaders was 0.191 points higher and this result was significant at the level of 5%, which was consistent with the original conclusion. We then used kernel matching and radius matching to ensure the accuracy of the results. The kernel matching and radius matching results were similar to those of nearest neighbor matching. Kernel matching and radius matching revealed that the self-rated health scores of retired danwei leaders increased by 0.172 and 0.186, respectively. The above results remain robust.

**Table 6:** Average treatment effect on the treatment group

Matching method	Treatment group	Control group	ATT	Bootstrap standard error	*T*-value
Nearest neighbor matching	3.485	3.293	0.191^a^	0.080	2.80
Kernel matching	3.485	3.312	0.172^b^	0.051	3.40
Radius matching	3.485	3.297	0.186 ^a^	0.080	2.73

^a^ and ^b^ indicate significance at 5% and 1%, respectively; the standard error after matching in line 5 is calculated by bootstrapping 1000 times

The above results show that compared with retirees of other occupational types and levels, retired danwei leaders had a more positive evaluation of their own health status. Considering the actual situation in China, we find that, on the one hand, unlike foreign leaders, Chinese danwei leaders can still maintain high social status even after they retire owing to their old social network. In other words, there is a special “inheritance” phenomenon among Chinese danwei. The successor continues to remain a part of the social network of the retired danwei leader to ensure that former leaders can still maintain a certain degree of control over power, which greatly weakens the sense of uselessness among retired danwei leaders. On the other hand, compared with retirees of other occupations, danwei leaders often have greater advantages in socioeconomic status. This occupational advantage can help danwei leaders for a long time through the accumulative effect, such that they can still enjoy the benefits of their previous occupation after retirement. This includes being able to maintain higher social status and obtain a higher income. This further enables them to still have the spiritual satisfaction and material security that are difficult to obtain in other occupations after retirement, thereby resulting in higher self-rated health level.

### Mechanism analysis

Based on the above analysis, compared with other occupations, danwei leaders can affect their post-retirement health levels through their social status and income. This shows that compared with non-danwei leaders, retired danwei leaders have both higher post-retirement incomes and higher social status, meaning they can still gain respect, exert influence, and obtain and provide social support even after retirement. Based on these possible influences and the availability of data, this study selected the total personal income of the respondents in the past year to measure their post-retirement incomes; further, we examined whether they had mediated disputes in the past year to assess their social status and analyze the possible mechanisms affecting the self-rated health of retired danwei leaders.

Specifically, this study used the stepwise regression method proposed by Wen et al. [52]. First, the independent variable was used to perform a regression for the mediating variable and then the mediating variable was added into the benchmark model for the regression analysis. Models 3 and 5 are the regression results of the mediating variables to being danwei leader before retirement, whereas Models 4 and 6 are the regression results of the benchmark model after adding each mediating variable. The regression results are shown in Table 7. As seen in Model 3, danwei leaders had a higher income after retirement. The results of Model 4 indicated that the coefficient of self-rated health for danwei leaders decreased when the post-retirement income variable was included. Meanwhile, post-retirement income was significantly and positively related to self-rated health scores. This suggests that post-retirement income is a mediating mechanism for the higher self-rated health of danwei leaders. Following the same steps to interpret Models 5 and 6, it was revealed that social status is another mediating mechanism by which being a danwei leader before retirement affects self-rated health after retirement. Therefore, serving as a danwei leader before retirement enhanced self-rated health after retirement through both post-retirement income and social status.

**Table 7:** Mediating effect estimation results

Variable	Model 3	Model 4	Model 5	Model 6
	lncome	Health	Mediation of disputes	Health
Occupation	0.808^c^	0.096^a^	0.441^b^	0.106 ^a^
	(0.066)	(0.048)	(0.128)	(0.048)
lncome		0.025 ^b^		
		(0.009)		
Mediation of disputes				0.140 ^c^ (0.027)
Control variables	yes	yes	yes	yes
N	5910	5910	5910	5910
r2/pr2	0.245	0.166	0.023	0.170

Robust standard errors are in parentheses; ^a^, ^b^, and ^c^ indicate significance at the 5%, 1%, and 0.1% levels, respectively

It should be noted that, first, Chinese danwei leaders can also maintain better welfare and living conditions after retirement, which makes them less likely to experience insecurity and anxiety due to quitting their jobs. Second, as one’s status in the social hierarchy affects one’s level of social participation [53], Chinese danwei leaders—with higher subjective social status assessments (i.e., individuals’ statuses relative to their peers)—have more advantages in this aspect. For example, the special succession mechanism in Chinese organizations and the traditional Chinese culture of respecting one’s superiors enable retired leaders to maintain influence and social status through old connections and thus reduce the sense of gap.

In conclusion, compared with other retirees, Chinese danwei leaders not only enjoyed material security after retirement but also had a reduced sense of loss through the continuation of their social status and influence, thus reducing their sense of uselessness and helplessness. All of these factors significantly helped them better self-rate their health after retirement.

### Heterogeneity analysis

The impact of being a danwei leader before retirement on an individual’s self-rated post-retirement health may be affected by personal characteristics and socioeconomic factors. In this regard, we further tested the difference in the impact of self-rated post-retirement health caused by being a danwei leader before retirement under different backgrounds such as the retiree’s sex and region. We regressed the sample in separate groups according to variables such as sex and region and then compared the effects of being a danwei leader in these different groups on self-rated health.

Table 8 reports the estimation results. It can be seen that in terms of sex, the self-rated health of female retired danwei leaders was higher than that of male leaders. Geographically, the self-rated health of retired danwei leaders in non-eastern provinces was higher than that in eastern provinces. In terms of marital status, retired danwei leaders with a spouse reported higher self-rated health than those without a spouse. Retired danwei leaders who did not live with their children also had a more positive evaluation of their own health than those who did live with their children. Finally, retired danwei leaders with higher levels of education gave a higher evaluation of their health than those with lower levels of education.

**Table 8:** Heterogeneity analysis

Variable	By sex			By region		By marriage		
	Male	Female		Eastern	Non-eastern	Married		Unmarried
Occupation	0.059	0.205^b^		0.086	0.138^a^	0.110 ^a^		0.176
	(0.060)	(0.078)		(0.076)	(0.062)	(0.053)		(0.110)
Control variables	yes	yes		yes	yes	yes		yes
N	2978	2932		2615	3295	4522		1388
r2	0.183	0.154		0.153	0.172	0.149		0.173
Variable	By living situation				By education			
	Living with children		Not living with children		Low education		High education	
Occupation	0.031		0.174 ^b^		0.101		0.126 ^a^	
	(0.081)		(0.059)		(0.084)		(0.059)	
Control variables	yes		yes		yes		yes	
N	2172		3738		3495		2415	
r2	0.190		0.144		0.137		0.189	

Robust standard errors are in parentheses; ^a^ and ^b^ indicate significance at the 5% and 1% levels, respectively

## Conclusions

Using data from the 2018 CLASS, this study examined the self-rated health of retired danwei leaders in China. The empirical results show that the occupation of a danwei leader before retirement in China had a positive impact on self-rated health after retirement. More specifically, the self-rated health score of retired danwei leaders increased by 0.117 points and was significant at the 5% level. Therefore, the results show that retired danwei leaders have a higher self-rated health level than retirees in other occupations. The accumulative effect of occupation plays a positive role in this, as retired danwei leaders can still enjoy the advantages of their previous occupation. Through the heterogeneity analysis, we confirmed that the positive effect of being a danwei leader before retirement on self-rated post-retirement health mainly existed in the older adult group with the following characteristics: female, from a non-eastern region, married, not living with children, and a high education level. Finally, through the mediating effect test, this study found that the influence of serving as a danwei leader on self-rated post-retirement health was mainly realized by the role of post-retirement income and social status.

Based on the above conclusions, to improve the self-health perception of retirees, this study puts forward the following suggestions. First, we should examine the fairness of the post-retirement welfare benefits of different occupational types and levels to avoid the physical and mental health problems of retirees with low occupational levels due to the inequality of welfare benefits. The government can provide equal medical security and certain subsidies to retirees with lower occupational grades and in less economically developed areas by focusing on their health needs and living standards. Second, we should pay attention to retirees’ inability to adapt to their new social identity and retirement life and reduce their sense of uselessness, powerlessness, helplessness, and loss. The government can mobilize retirees to participate in social activities and alleviate their sense of social isolation and loss caused by retirement. Moreover, they can promote the establishment of improved social infrastructure, provide environments for retirees to engage in physical activities, and encourage them to establish good living habits and find new life pursuits. Third, we should consider any differences in sex, region, and family status. Compared with women, retirement increases men’s depression and leads to a greater decline in their mental health, which has a significant negative impact on their overall health [54]. The same is true for male danwei leaders. Therefore, it is necessary to pay special attention to the mental health status of male danwei leaders after retirement. For danwei leaders in the eastern region, the government should ensure that their retirement income is commensurate with the higher cost of living in the east and reduce their financial insecurity after retirement. Similarly for danwei leaders without spouses, the government should promote their participation in social activities, reduce their loneliness, and provide them with certain life care. Fourth, we should focus on the retirement activities of different occupational groups. Different occupational groups are encouraged to choose appropriate pension plans according to their personal conditions. For example, according to their family situation and income level, they can choose family pensions, independent pensions, and collective community pensions, among others. In contrast, the government should provide the necessary technical, financial, policy, and talent support for retirees’ pension activities. The government needs to promote and improve the construction of older adult care facilities, encourage social forces to participate in voluntary services for older adults, strengthen the training of talent in older adult care services, and build a professional older adult care system for different occupational groups.

This study has certain limitations. First, owing to limited data, only cross-sectional data were used. We did not use panel data, which might have strengthened the causal interpretation. In future studies, we suggest using suitable panel data, if available, to validate the findings of this study. Second, this study only controls for observable selection bias. There may also be unobservable selection bias. To some extent, this also stems from the problem of data availability and lack of appropriate instrumental variables on the occupational stratification in the available data from Chinese social surveys. Therefore, we only controlled for observable selection bias using the PSM method; future studies should control for unobservable selection bias. Third, the mechanism analysis needs to be further explored. In this study, we explored two mediating mechanisms: post-retirement income and social status. However, many interesting mediating mechanisms, such as lifestyle habits and health care resources, are yet to be explored. Fourth, owing to data limitations, we did not control for health status in early life. However, health status in early life not only affects whether an individual becomes a danwei leader, but also influences health status after retirement, biasing the final estimation results. Subsequent studies should aim to address the estimation bias caused by health status in early life. These limitations highlight the need for further research and should be addressed in future studies.

## Data Availability

The datasets generated and/or analysed during study can be found in the CLASS [http://class.ruc.edu.cn/].

## List of abbreviations

ATT: Average treatment effect on the treatment group
CLASS: China Longitudinal Aging Social Survey
OLS: Ordinary least squares
PSM: Propensity score matching
